# Orthorexia Nervosa, Eating Disorders, and OCD: A Literature Review and Diagnostic Challenges

**DOI:** 10.1192/j.eurpsy.2025.705

**Published:** 2025-08-26

**Authors:** M. A. Rodrigues, F. M. Amorim, M. H. R. Delgado, L. A. Lemes

**Affiliations:** 1 Universidade Anhembi Morumbi, São Paulo, Brazil

## Abstract

**Introduction:**

Orthorexia Nervosa (ON) is characterized by an obsessive focus on healthy eating, leading to extreme restrictions and negative impacts on mental and social health. ON shares traits with eating disorders (EDs) and obsessive-compulsive disorder (OCD), but its diagnostic status remains unclear. Recent studies suggest ON may be linked to personality traits like perfectionism, common in both EDs and OCD (3,5). The COVID-19 pandemic has exacerbated these issues, stressing the need for better understanding of ON and its overlaps with other disorders (6).

**Objectives:**

This study reviews literature from the past seven years to explore the connections between ON, EDs, and OCD. It examines ON as a possible distinct diagnosis, focusing on the role of personality traits like perfectionism and evaluates the clinical implications and treatment needs, considering cultural and contextual factors.

**Methods:**

A systematic review was conducted using PubMed, PsycINFO, and Scopus, covering studies from 2017 to 2023. The studies included clinical and non-clinical samples, focusing on psychopathological features and personality traits linked to ON, EDs, and OCD (1-2). The review analyzed diagnostic criteria, prevalence, personality trait correlations, and the impact of contextual factors like the COVID-19 pandemic.

**Results:**

ON has a complex relationship with EDs and OCD. In ED patients, especially those with anorexia nervosa (AN) and bulimia nervosa (BN), orthorexic behaviors were more prevalent, suggesting ON might be a subtype of EDs rather than a separate condition. ON scores in ED patients decreased after targeted treatment, supporting this connection (1). Personality studies showed that orthorexic individuals often display perfectionism, particularly concerns about mistakes and unrealistic standards. These traits are transdiagnostic, affecting ON, EDs, and OCD (2-3). ON was also linked to specific personality profiles, such as paranoid and narcissistic traits, which worsen symptoms and complicate treatment (2). ON symptoms were strongly correlated with anxiety, depression, and ED and OCD symptoms, indicating shared psychopathological mechanisms (5). The ego-syntonic nature of ON, where behaviors are seen as normal, may lead to underdiagnosis and reduced treatment-seeking (5). The COVID-19 pandemic further exacerbated orthorexic behaviors, highlighting the need for tailored clinical interventions for at-risk individuals (6).

**Conclusions:**

ON should be viewed within the ED spectrum, with significant overlaps with OCD and influence from traits like perfectionism. Cultural and definitional differences complicate its classification as a distinct disorder. The COVID-19 pandemic has highlighted the clinical importance of ON and the need for effective prevention and treatment strategies. Future research should aim to develop standardized diagnostic criteria and explore personality traits to improve ON diagnosis and treatment.

**Disclosure of Interest:**

None Declared

